# Genotype-specific relationships among phosphorus use, growth and abundance in *Daphnia pulicaria*

**DOI:** 10.1098/rsos.170770

**Published:** 2017-12-13

**Authors:** Ryan E. Sherman, Priyanka Roy Chowdhury, Kristina D. Baker, Lawrence J. Weider, Punidan D. Jeyasingh

**Affiliations:** 1 Department of Integrative Biology, Oklahoma State University, Stillwater, OK 74078, USA; 2 Department of Biology, Program in Ecology and Evolutionary Biology, University of Oklahoma, Norman, OK, USA

**Keywords:** ecological stoichiometry, growth rate, intraspecific competition, phenotypic plasticity, phosphorus, zooplankton

## Abstract

The framework ecological stoichiometry uses elemental composition of species to make predictions about growth and competitive ability in defined elemental supply conditions. Although intraspecific differences in stoichiometry have been observed, we have yet to understand the mechanisms generating and maintaining such variation. We used variation in phosphorus (P) content within a *Daphnia* species to test the extent to which %P can explain variation in growth and competition. Further, we measured ^33^P kinetics (acquisition, assimilation, incorporation and retention) to understand the extent to which such variables improved predictions. Genotypes showed significant variation in P content, ^33^P kinetics and growth rate. P content alone was a poor predictor of growth rate and competitive ability. While most genotypes exhibited the typical growth penalty under P limitation, a few varied little in growth between P diets. These observations indicate that some genotypes can maintain growth under P-limited conditions by altering P use, suggesting that decomposing P content of an individual into physiological components of P kinetics will improve stoichiometric models. More generally, attention to the interplay between nutrient content and nutrient-use is required to make inferences regarding the success of genotypes in defined conditions of nutrient supply.

## 1. Introduction

The framework of ecological stoichiometry (ES) [1] uses the elemental composition of species to make predictions about the growth and competitive ability of a given species in defined environmental supply conditions. Species differ in stoichiometry considerably [2]. Such differences in elemental demand alter relative growth rates of species under constant conditions of supply stoichiometry (e.g. [3–5]). Further, variation in key life-history traits underlies such differences in demand [6]. For example, Iwabuchi & Urabe [7] conducted microcosm experiments on three *Daphnia* species and found that those with low phosphorus (P) demand, species of the freshwater cladoceran genus *Daphnia*, outcompeted others with a high P-demand, under P-limiting conditions. Their results indicated direct effects of supply stoichiometry in determining the growth of populations varying in elemental demand. Within plants, Yu *et al*. [8] analysed long-term data in several vascular plants and found that the degree of stoichiometric homeostasis in the face of variable supply stoichiometry (as opposed to elemental content in a single supply environment), best predicts the abundance of a plant species.

While there are considerable interspecific differences in elemental content, there is growing evidence that intraspecific (i.e. within population) variation can be considerable as well (e.g. [9–14]). Our understanding of the processes generating and maintaining such variation, and associated ecological consequences is sparse. Testing the extent to which stoichiometric models built to predict the success of species' performance when extended to genotypes within a species should illuminate important processes that maintain substantial intraspecific variation in stoichiometry.

Intraspecific variation in stoichiometry has to be driven by some combination of differential acquisition, assimilation, net incorporation or retention of elements [15]. Functional genomic analyses of *Daphnia* genotypes within a species reveal substantial differences in gene expression that could underlie variation in stoichiometry [16]. Multiple genes have been identified to be involved in P homeostasis and that variation of P supply affects the expression of genes involved in P handling (e.g. phosphatases, P transporters; reviewed in Jeyasingh & Weider [17]). Note that substantial genetic variation exists in the expression of such genes in plants (e.g. tomato [18]; maize [19]; bean [20]) although little is known in animals. P supply has been shown to significantly alter the expression of approximately 20% of the genes in the *Daphnia* genome (e.g. [21]). Genotypic comparisons of transcriptomes revealed that genotypes differed in the expression of as much as 30% of the genes in common gardens [16,22]. Differential expression of genes and pathways could alter P allocation to biochemicals (e.g. phospholipids versus sulfolipids [23–25]), tissues (e.g. soft tissue versus carapace [26]), or life-history traits (e.g. growth versus reproduction [27]), which are known to differ in P-demand. While substantial genetic variation in life-history traits is well known [28,29], it is likely that similarly strong genetic variation exists for P allocation to biochemicals and tissues (e.g. bean [30]; wheat [31]; rice [32]; fish [33]). As such, two genotypes can have identical P content, and yet differ substantially in P allocation at the biochemical or tissue levels. Such differences could be manifested in the rates at which P is acquired, assimilated and excreted. Thus, it is possible that fitness-relevant classical traits (e.g. intrinsic rate of natural increase) can be expressed and maintained in a genotype by altering elemental kinetics without any change in individual body stoichiometry, and yet this results in distinctive competitive or fitness outcomes under the same supply stoichiometry.

We addressed these issues in the freshwater crustacean *Daphnia pulicaria* by testing the extent to which %P predicted growth rate and competitive ability (outcome of intraspecific competition), as well as measured the ^33^P kinetics (i.e. acquisition, assimilation, incorporation, retention) in high and low P supply. There is substantial intraspecific variation in P content (e.g. [9,11]) and physiological kinetics of P in *Daphnia* (e.g. [22]). This variation can interact with the P supply environment and affect the relative performance of genotypes [34–36]. The existence of intraspecific variation in P kinetics is perhaps not surprising, given that P supply alters the expression of about a third of the genes in the *Daphnia* genome [21]. For example, although genotypes did not differ in somatic P content, Frisch *et al*. [37] found significant differences in P use of *Daphnia* genotypes that had been hatched from resting eggs, separated temporally by centuries. Specifically, these ‘resurrected’ genotypes differed by as much as 200% in P-retention efficiencies (RetE), and by as much as 300% in the amount of biomass produced per mg P in the body under P-limiting conditions (phosphorus use efficiency; PUE). Such differences in P use, without any change in P content, had predictable impacts on the quantity and quality of algae via recycling by these daphniid grazers [38].

Based on stoichiometric models built to describe performance of species differing in somatic stoichiometry [1], we predicted that genotypes with lower P content will exhibit slower growth rate and be competitively superior in conditions of lower P supply. Moreover, based on the growth rate hypothesis [6], we predicted that P content should be correlated with growth, which is strongly related to competitive ability in *Daphnia* [39]. Further, using physiological models [40], we predicted that all genotypes will increase rates of P acquisition, assimilation and retention under lower P supply conditions. In addition, we predicted that genotypes with lower P content will acquire, assimilate and retain P at a slower rate compared to genotypes with higher P content. Finally, we tested the impact of using multi-proxy measures of P kinetics in forecasting genotypic success (growth and competition) using multivariate approaches coupled with model reduction criteria.

## 2. Material and methods

### 2.1. Experimental organisms

The green alga, *Scenedesmus obliquus*, served as the food resource in our experiment. It was grown in continuous-flow chemostats in COMBO media containing concentrations of either high phosphorus (HP 50 µmol l^−1^) or low phosphorus (LP 5 µmol l^−1^) [41] at 20°C and constant light (approx. 120 µmol m^−2^ s^−1^). These P supply conditions produced algae with a C : P ratio of approximately 150 (HP) and approximately 750 (LP), respectively. *Daphnia pulicaria* resting eggs were isolated from the 20–24 cm sediment layer (sediment dated between *ca* 1967 and 1977 AD) of South Center (SC) Lake, Chisago County, Minnesota (for collection details see Frisch *et al*. [37]) and propagated parthenogenetically in the laboratory. *Daphnia pulicaria* clonal lineages were stored in a temperature-controlled growth chamber at 20°C with an 18 L : 6 D cycle in COMBO media containing no N or P [41]. Ten genetically distinct genotypes from this resurrected population were used in this experiment, each possessing a unique electromorph marker at the glucose-6-phosphate isomerase (GPI; Enzyme Commission (EC) 5.3.1.9) locus and/or phosphoglucomutase (PGM; EC 5.4.2.2) locus.

### 2.2. Elemental analysis

To determine algal stoichiometry, *Scenedesmus* was filtered from experimental chemostats onto pre-combusted (550°C for 2 h) and pre-weighed GF/C filters (Whatman, Maidstone, UK), and dried at 60°C for 72 h. Carbon (C) content was determined using an automated CHNOS analyzer (VarioMICRO analyzer, Elementar Americas, NJ, USA). Total phosphorus (P) content was quantified by a modified sulfuric acid digestion method (APHA 1992) and verified using a spinach standard (NIST 1570a). We then converted C and P content by mass into molar C:P for analyses.

To determine body phosphorus content in *D. pulicaria* raised in contrasting P environments, we raised neonates (less than 24 h old) individually in jars in 100 ml of COMBO media containing no N or P [41]. Individuals were fed 1 mg C l^−1^ per day of either HP or LP *Scenedesmus* algae for a period of 5 days. The culture media was replaced each day. P content of 10 different genotypes was measured following a 5-day period under either HP or LP conditions, with three replicates per treatment. To determine *D. pulicaria* P content, pre-weighed samples were combusted (550°C for 2 h) and dried at 60°C for 72 h. Total phosphorus (P) content was quantified by a modified sulfuric acid digestion method (APHA 1992) and verified using a spinach standard (NIST 1570a). Each sample was a pooled sample consisting of approximately 0.05 mg. P content was calculated as per cent of *Daphnia* dry mass.

### 2.3. Radiotracer assays

#### 2.3.1. Radiolabelling algae

Radioisotope assays using ^33^P allowed us to examine elemental use on a per-atom basis. After ingestion by an organism, we were able to measure the quantity of radioisotopes acquired by the organism. Inorganic radiotracers are primarily introduced to consumers via ingestion of autotrophic periphyton [42]. Therefore, our radiotracer assays provided a robust test of ingestive and post-ingestive P processing. To introduce the radioisotopes into the *Scenedesmus* algae, fresh algae, both HP and LP, were centrifuged at 3600 rpm for 30 min. The supernatant was discarded and the precipitate was resuspended in 100 ml of no N and P COMBO medium [41]. We then added 5.55 Mbq of ^33^P (as orthophosphate) to each 100 ml algal sample. The solutions were kept on a shaker at 20°C, 16 L : 8 D cycle and incubated for 72 h to allow the algae to fully incorporate the radioisotope. *Scenedesmus* algae was assumed to be uniformly radiolabelled. Following 72 h, the algal solution was centrifuged at 3600 rpm for 30 min, after which the supernatant was discarded and the precipitate was resuspended in 100 ml of no N and P COMBO medium [41]. Two millilitres of the radiolabelled algae was then filtered through a GF/F filter (Whatman International Ltd, Maidstone, UK). Following an addition of scintillation cocktail (2 ml; Ultima Gold, PerkinElmer Inc., MA), specific activity was counted in a liquid scintillation counter (Beckman Coulter LS 6500).

#### 2.3.2. ^33^P Kinetics

We performed ^33^P radiotracer assays, following DeMott, Gulati and Siewertsen [43] and Roy Chowdhury *et al.* [22]. Similarly sized, approximately 5-day-old *D. pulicaria* were isolated in no N and P COMBO and acclimated to experimental conditions by feeding on 1 mg C l^−1^ of either HP or LP *Scenedesmus* algae daily for 72 h. On the day of the experiment, *Daphnia* were pooled in jars of 5, with a minimum of 3, maximum of 5 replicates and starved for 2 h. Following starvation, the *Daphnia* were fed 1 mg C l^−1^ radiolabelled algae, either HP or LP*.* It is important to note that ^33^P is a small portion of the total P in the algae; however, it captures general patterns in P processing. Additionally, not all the same animals could be used to evaluate P kinetics. Four different rates were calculated: acquisition (feeding *Daphnia* radiolabelled algae for 10 min), assimilation (40 min of feeding and 6 h of gut clearing), net incorporation (4 h of feeding) and retention (10 min of feeding and 12 h of gut clearing). When measuring retention, we changed the media every 1, 2, 4 and 8 h in order to prevent recycling. We defined acquisition as the intake of P in a given period (10 min), before having been absorbed through the gut wall. Assimilation was defined as absorption of those elements through the gut wall into body tissue, net incorporation as the amount of P allocated following assimilation, and retention as the amount of P retained after 12 h of not feeding. *Daphnia* thus collected were placed in scintillation vials for radioactive counting (Beckman Coulter LS 6500). The amount of radioactive ^33^P was calculated in each organism, correcting for mass in micrograms per microgram dry weight from an average of five separate individuals raised in identical conditions. All samples for each genotype were taken and analysed on the same day of the experiment.

### 2.4. Growth rate

To determine juvenile growth rate under contrasting P supply conditions, *D. pulicaria* individuals were fed 3 mg C l^−1^ per day (to elucidate greater differences in growth) of either HP or LP *Scenedesmus* algae, with four replicates per treatment. The culture media was replaced daily. Growth rates of 10 different genotypes were measured over a 5-day period under either HP or LP conditions. Four neonates of each genotype (less than 24 h old) were used for each P-supply treatment, separated individually in 100 ml of COMBO media containing no N or P [41]. The length of each neonate in millimetres was measured from the top of the head to the base of the tail-spine on the first and fifth (final) day of the experiment using a microscope ocular micrometre at 4× magnification (Leica S8APO, Leica Microsystems, IL, USA). Growth rate was determined as the change in length per day from birth to day 5. Note that growth rate was also estimated as change in length per day per initial length, to scale for variation in the starting size of individuals. Both approaches showed no differences in any statistical analyses, therefore, all analyses were performed with growth rate defined as change in length per day from birth to day 5.

### 2.5. Competitive ability

**Figure 1.**
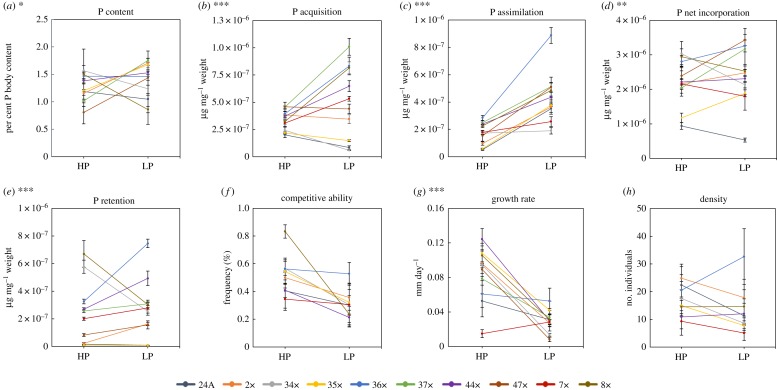
Univariate responses to P supply conditions in 10 genotypes of *D. pulicaria*. Phosphorus treatment is along the *x*-axis and phenotypic trait measured along the *y*-axis (P content (*a*), P kinetics (*b*–*e*) and fitness-relevant traits (*f–h*)). HP, high phosphorus; LP, low phosphorus. The symbol * indicates a significant genotype-by-treatment interaction with (*a*) *p* < 0.01, (*d*) ** indicates *p* < 0.001 and (*b,c,e,g*) *** *p* < 0.0001.

Six neonates (less than 24 h old) from each genotype were placed in competition with six neonates of a reference genotype. Three reference genotypes were chosen from the 4–8 cm sediment layer (sediment dated between *ca* 2002 and 2008 AD) from SC Lake, and were used to compare competitive abilities of the experimental genotypes from the 20–24 cm layer of SC. Each reference genotype was paired with each experimental genotype for a total of 30 trials per P treatment. Neonates were placed in 500 ml flow-through polyvinyl chloride microcosms containing no N and P COMBO medium [41]. The flow-through chamber microcosms were modelled after Heugens *et al*. [44] and allowed for a controlled supply of food and nutrients, while eliminating the loss of algae from the water column by sedimentation. *Scenedesmus obliquus* algae were pumped into microcosms one hour before adding neonates, so as to have food available upon immediate transfer. Each 500 ml microcosm vessel received 1 mg C l^−1^ per day of either HP or LP algae continuously for a duration of 21 days at a rate of approximately 1.725 l day^−1^. There was no early mortality within the first 5 days. Following the 21 days, microcosm chambers were homogenized and 30% (150 ml) of the media was sampled. Twelve *Daphnia* were chosen at random from a 50 ml subset of the 150 ml sample to test for unique genotype-specific (allozyme) markers. Allozyme electrophoresis followed the protocols of Hebert & Beaton [45], and allowed us to estimate the frequency of each experimental *Daphnia* genotype in competition. Each genotype possessed a unique allozyme (i.e. electromorph) marker at the glucose-6-phosphate isomerase (GPI; Enzyme Commission (EC) 5.3.1.9) locus and/or PGM (EC 5.4.2.2) locus. Two experimental genotypes (from 20–24 cm sediment layer) share the same GPI electromorph profiles with two of the reference genotypes (from the 4–8 cm sediment layer). Distinguishing these three experimental genotypes from the reference genotypes under competition proved equivocal using the PGM (EC 5.4.2.2) locus. Therefore, these two genotypes were excluded from analysis involving competitive ability. By identifying the frequency of each experimental genotype relative to the reference genotypes, we compared the competitive abilities of the experimental genotypes under varying P-supply treatments. Experimental genotype frequencies were calculated by dividing the number of individuals of the target genotype by the total number of individuals (experimental and reference) at the conclusion of the experiment. Estimated density values were calculated from the fraction of individuals of the target genotype (out of the 12 sampled) multiplied by the total number of individuals in each microcosm following the 21-day period. Estimated density values are represented in figure 1 and table 1.

**Table 1.** Univariate responses of 10 *D. pulicaria* genotypes to contrasting P supply conditions. Bold font indicates significant results with *p* < 0.05.

	genotype (G)			treatment (T)			G × T		
GLMM	*F*	d.f.	*p*	*F*	d.f.	*p*	*F*	d.f.	*p*
per cent phosphorus body content	0.596	8	0.760	3.542	1	0.097	3.164	8	**0**.**008**
P acquisition	2.178	9	0.131	3.190	1	0.108	26.951	9	<**0**.**0001**
P assimilation	2.341	9	0.111	26.944	1	**0**.**001**	10.203	9	<**0**.**0001**
P net incorporation	4.348	9	**0**.**020**	0.590	1	0.462	3.687	9	**0**.**001**
P retention	2.607	9	0.085	0.147	1	0.711	26.02	9	<**0**.**0001**
juvenile growth rate	0.829	9	0.608	21.396	1	**0**.**001**	5.505	9	<**0**.**0001**
competitive ability	0.927	7	0.539	9.314	1	**0**.**018**	0.247	7	0.545
density	2.728	7	0.104	1.408	1	0.274	0.985	7	0.460

### 2.6. Statistical analysis

To test the interactive effects of *Daphnia* genotype and P supply on P content, P kinetics (acquisition, assimilation, net incorporation and retention), growth rate, competitive ability, we used separate general linear mixed models (GLMMs) with *Daphnia* genotype as a random effect. All non-normal data were log-transformed for analyses to fit assumptions for GLMMs. Principal component analysis (PCA) was implemented as a dimension reduction technique to visualize our multivariate data (i.e. growth rate, P content, P kinetics, competitive ability). The PCA was run on a correlation matrix. Correlation matrix PCAs centre and standardize the variables so variables with large values and thus higher chance of variation do not have outsized influences on multivariate patterns. To improve interpretability of the components extracted by the PCA, we applied an orthogonal rotation (varimax) to the components, reporting PC axes with an eigenvalue greater than one. For all traits measured, we did not have an equal number of replicates; therefore, we excluded missing pairwise values for the multivariate analysis (see electronic supplementary material, table S1 for more details). PC axes scores were plotted to visualize the general relationships between all traits measured in a two-dimensional space. Linear regressions and analyses of covariance (ANCOVA) were run on P content, growth rate and competitive ability to test predictions based on ES, i.e. that P content and growth rate should be positively correlated, that growth rate predicts competitive ability, and that P content predicts competitive ability. All tests were performed using SPSS (IBM Statistics v. 22).

A model comparison approach was used to evaluate competing models to explain growth rate and competitive ability in the *Daphnia* genotypes. All model comparison data analyses were conducted using the R statistical package MuMIn (R Core Team 2015, version 3.2.3) to measure the relative performance of the models according to Akaike information criterion corrected for small sample sizes (AICc). *Daphnia* P-use variables were included in the full model as potential predictors, with the model comparison approach used to determine their relative importance to growth and competitive ability. These models were analysed using model selection based on AIC model selection approach to determine which traits best explained growth rate and competitive ability. Using the ΔAICc scores and AICc weights, the top candidate models were identified by removing models with ΔAICc scores greater than 6. Generalized linear models were used to compare a set number of models to the data, measuring the relative support the data gave to each model.

## 3. Results

### 3.1. Sources of intraspecific variation

Reaction norm plots (figure 1) of the *D. pulicaria* genotypes showed varied responses to P supply conditions with a significant genotype × P treatment interaction. There was a significant genotype-by-treatment interaction for *Daphnia* %P (*p* = 0.008), P acquisition, retention and assimilation (*p *< 0.0001), and P net incorporation (*p* = 0.001) (figure 1 and table 1).

Juvenile growth rate showed a significant genotype by treatment interaction (*p* < 0.0001; figure 1*g* and table 1). There was no genotypic variation in competitive ability, though there was a significant treatment effect (*p* = 0.018; figure 1*f* and table 1). There were no significant genotypic or treatment effects on experimental clone density (figure 1*h* and table 1). There were also no differences in competitive ability among the reference genotypes.

### 3.2. Relationships among P content, P kinetics, growth and competitive ability

**Figure 2.**
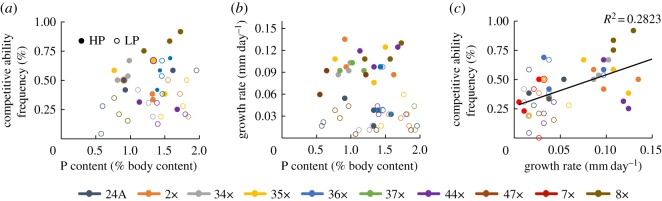
Linear regressions testing predictions based on ES. Linear relationship between (*a*) somatic P content and competitive ability; (*b*) somatic P content and growth rate; and (*c*) growth rate and competitive ability. Open circles represent low phosphorus (LP) treatments; solid circles represent high phosphorus (HP) treatments. Competitive ability of genotypes was not correlated with somatic P content (*p* = 0.267; *R*^2^ = 0.01). P content was not significantly correlated with growth rate (*p* = 0.144; *R*^2^ = 0.06). Competitive ability was significantly correlated with growth rate (*p* < 0.0001; *R*^2^ = 0.28).

**Figure 3.**
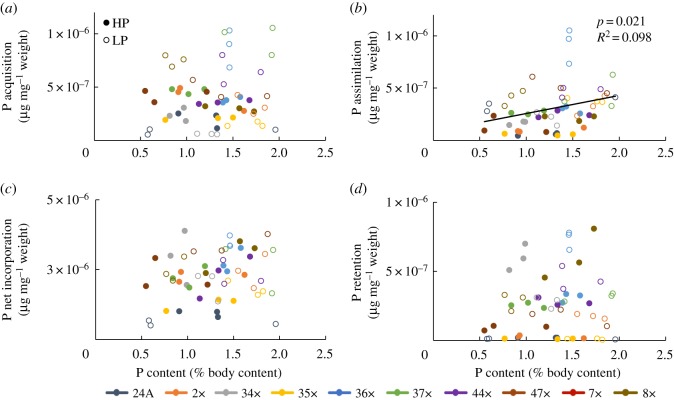
P content and P-use linear relationships, (*a*) P acquisition, (*b*) P assimilation, (*c*) P net incorporation (*d*) P retention. Open circles represent low phosphorus (LP) treatment; solid circles represent high phosphorus (HP) treatment.

Stoichiometric models predict that individuals with lower somatic P content will be competitively superior under LP conditions [1]. Competitive ability of genotypes was not correlated with somatic P content (*p* = 0.267; *R*^2^ = 0.006; figure 2*a*). ANCOVA results revealed that P content and competitive ability did not covary (*p* = 0.146). P content and competitive ability were not significantly correlated, when separated by treatment (HP, *p* = 0.917; LP, *p* = 0.072). The growth rate hypothesis predicts that rapid growth should be correlated with high somatic P content [6]. However, P content was not significantly correlated with growth rate (*p* = 0.144; *R*^2^ = 0.041; figure 2*b*). ANCOVA results showed that P content and growth rate covaried (*p* = 0.002). Faster growth rate predicts higher competitive ability due to fitness advantages related to size. Linear regressions revealed that competitive ability was significantly correlated with growth rate (*p* < 0.0001; *R*^2^ = 0.28; figure 2*c*). Growth rate and competitive ability were not significantly correlated when separating by treatment (HP, *p* = 0.07; LP, *p* = 0.25). P content was not correlated with P acquisition (*p* = 0.412; *R*^2^ = 0.013; figure 3*a*) nor with P net incorporation (*p* = 0.111; *R*^2^ = 0.048; figure 3*c*) or P retention (*p* = 0.357; *R*^2^ = 0.016; figure 3*d*). P content was significantly correlated with P assimilation (*p* = 0.021, *R*^2^ = 0.098, figure 3*b*).

**Figure 4.**
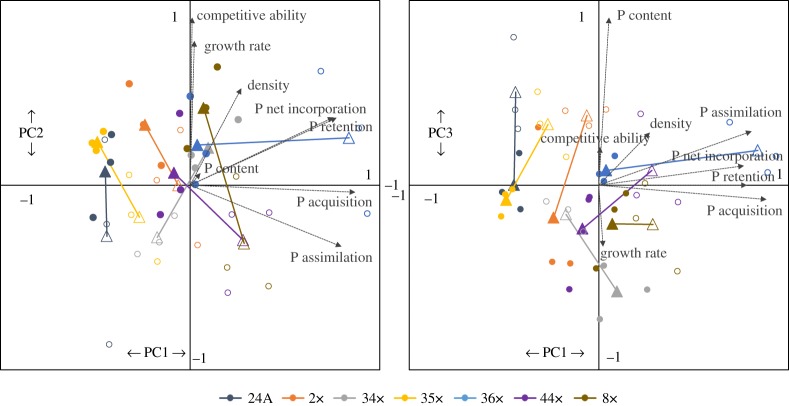
PCA plots showing as solid lines, the phenotypic trajectories of *D. pulicaria* genotypes (multivariate response to P supply conditions for P content, P kinetics, growth rate and competitive ability traits). PC1 plotted on the *x*-axis against (*a*) PC2 and (*b*) PC3 on the *y*-axis. Open shapes represent low phosphorus (LP) treatment and solid shapes represent high phosphorus (HP) treatment. Circles are the raw data values, while triangles represent centroids. HP and LP centroids are connected by a line to show the directionality between HP and LP conditions. Percentage of the total variation explained by the first three axes of the PCA was approximately 74%. P-use traits explained the most variation on PC1, explaining approximately 34% of the total variation, with PC2 explaining approximately 26% of the variation and PC3 explaining approximately 15%.

The individual loadings on the first three axes of the principal component analysis (PCA) of each dataset, summary of variance explained by the PCA, and eigenvalues for each axis can be found in electronic supplementary material, table S2. Seven of the 10 genotypes are reported in the PCA; one genotype had incomplete P content data and two remaining genotypes shared the same GPI electromorph profiles with two of the reference genotypes. As previously stated, distinguishing these two experimental genotypes from the reference genotypes proved equivocal using the PGM (EC 5.4.2.2) locus and, therefore, these two genotypes were excluded from the multivariate analysis. Percentage of the total variation explained by the first three axes of the PCA was approximately 74%. P-use traits explained the most variation on PC1, explaining approximately 34% of the total variation; PC2 explaining approximately 26% and PC3 explaining approximately 15% (figure 4). Plotting phenotypic trajectories revealed differences in the nature and magnitude of change between genotypes in multivariate space in response to altered P supply conditions. In response to LP conditions, genotypes tended to decrease along PC2, representing a decrease in growth rate, competitive ability and density (figure 4*a*). Genotypes increased along PC1 to varying degrees in response to LP conditions, thus, representing an increase in P acquisition and assimilation as a response to limited P-supply conditions. Notably, clone 36x did not differ in growth rate between P supply treatments, as it increased its P acquisition and assimilation under LP (figure 4*a*).

### 3.3. Model comparison

The best supported model for competitive ability included P assimilation and P retention (table 2). The best supported model for growth rate included P acquisition, P assimilation and P retention (table 3). With competitive ability as the dependent variable, P content only appeared in one of the top five models (out of 20 models) and in none of the top five models when growth rate was the dependent variable (out of 13 models). P assimilation was in all of the top five models for predicting both competitive ability and growth rate. P assimilation has the highest relative importance, while P content has the lowest for both competitive ability and growth rate (table 4). See model selection tables with competitive ability (electronic supplementary material, table S3) and growth rate (electronic supplementary material, table S4) for more information.

**Table 2.** Top five models for predicting competitive ability.

variables contained in model					d.f.	log lik	AICc	ΔAIC	weight
	P assim.		P ret.		4	2.592	3.9	0.00	0.231
	P assim.	P net.			4	2.064	5.0	1.06	0.136
	P assim.	P net.	P ret.		5	3.262	5.1	1.24	0.124
	P assim.		P ret.	P content	5	2.696	6.3	2.38	0.070
P acq.	P assim.		P ret.		5	2.618	6.4	2.53	0.065

**Table 3.** Top five models for predicting growth rate.

variables contained in model				d.f.	log lik	AICc	ΔAIC	weight
P acq.	P assim.		P ret.	5	84.076	−156.5	0.00	0.230
	P assim.		P ret.	4	82.483	−155.9	0.06	0.170
	P assim.	P net.		4	81.988	−154.9	1.59	0.104
	P assim.	P net.	P ret.	5	83.272	−154.9	1.61	0.103
P acq.	P assim.	P net.		5	82.987	−154.3	2.18	0.077

**Table 4.** Relative importance of the explanatory variables (P use and P content) for predicting growth rate and competitive ability. Number of models in which each variable was represented, for the top models with ΔAIC ≤ 6.

	P acq.	P assim.	P net.	P ret.	P content
growth rate as dependent variable					
relative importance (ΔAIC ≤ 6)	0.51	1.00	0.46	0.74	0.21
contained in top 13 models	7	13	8	8	6
competitive ability as dependent variable					
relative importance (ΔAIC ≤ 6)	0.23	0.79	0.53	0.67	0.20
contained in top 20 models	8	10	12	12	7

## 4. Discussion

The results from this study detail how P acquisition, assimilation and excretion (i.e. P kinetics) explain variation in growth rate and competitive ability when compared with P content alone in a population of *D. pulicaria*. Genotypes varied in plastic responses in P content and P kinetics to contrasting P supply conditions, differing in their nature and magnitude of plasticity (figure 1*a–e*). Under P limitation (i.e. LP conditions), P acquisition and assimilation increased, and exhibited more variation among genotypes when compared with HP conditions (figure 1*b,c*). P net incorporation and P retention varied significantly between HP and LP treatments (figure 1*d,e*). Although growth rates and competitive abilities varied among genotypes (figure 1*g*), these differences were not correlated with P content or P kinetics (figure 2). P content was significantly correlated with P assimilation, but no relationships were found between P content and P acquisition, net incorporation or P retention (figure 3). Genotypes varied in multivariate space, with most genotypes altering growth between high and low P conditions, while one genotype exhibited no difference in growth that was achieved by altering P kinetics (figure 4). P-kinetic traits explained the most variation on PC1, while competitive ability, growth rate and density explained the most variation along PC2, and P content explained the most variation along PC3 (figure 4*b*).

While most genotypes grew faster under HP conditions, this did not correspond to higher P content. We predicted that genotypes with lower P content would exhibit slower growth and be successful under LP conditions. Under LP conditions, some genotypes had an increase in P-use traits, likely to compensate for P deficiencies; i.e. demand of P for RNA biogenesis under the limiting conditions. Additionally, it may be possible that some genotypes can create more biomass with less P body content (i.e. differential PUEs), e.g. [37]. There was less variation in P acquisition under replete P conditions than under P-limited conditions (figure 1*b*). This suggests that under stressful, LP conditions, trade-offs between traits that require a sufficient amount of P are manifested. Large interspecific differences in P content are perhaps important in generating the robust growth–phosphorus couplings [46]. It is worth noting that even studies on species from the same genus do not show support for these predictions [47]. At the intraspecific level, where variation in P content is relatively more constrained, it is evident that growth may be sustained by other mechanisms that do not necessarily result in differences in P content. Differences in gene expression and P-use physiology [23–26] should underlie such variation, and represent an important area of further research.

In addition to variable strategies to achieve similar growth values, genotypes appear to use different strategies to achieve similar competitive outcomes against a set of common reference genotypes. Faster-growing individuals were found at higher frequencies in the competition experiment (*p* < 0.0001; *R*^2^ = 0.27; figure 2*c*), consistent with previous studies (e.g. [39]). While there were significant differences in response to changes in environmental conditions (variation in plastic response to P supply conditions) for the P-use traits, P body content and growth rate, these did not translate into variation in competitive ability among the *Daphnia* genotypes. The reference genotypes (from the 4–8 cm sediment layer) always outcompeted the experimental genotypes (20–24 cm sediment layer) in our study, a result that has been demonstrated previously in *D. pulicaria* genotypes [48]. However, genotypic variation in stoichiometry as a result of environmental supply of essential elements can potentially result in differential success. A few studies have reported striking shifts in the frequency of *Daphnia* genotypes depending on nutrient supply stoichiometry [11,34]. Weider *et al.* [34] showed that specific *Daphnia pulex* genotypes dominate under certain P supply conditions (C : P ∼ 100) versus others (C : P ∼ 800), demonstrating the large effect P supply can have on competitive outcomes in *Daphnia* populations. However, such competitive outcomes were probably related to other traits not directly related to P content [11]. It is likely that while P content has been useful in predicting the success of species, generating predictions on the success of genotypes is more complex.

P-use traits (i.e. P acquisition, assimilation, net incorporation and retention) are better at predicting growth rates and competitive abilities than P content alone (tables 2 and 3). It is not surprising that a multivariate approach is a better predictor of genotypic performance, when compared with P content alone (table 4). Nevertheless, using P content alone to make evolutionary inferences, as can be done at the interspecific level (e.g. [49]), could be misleading at the intraspecific level, and perhaps also over longer evolutionary time scales (e.g. [50]). Genotypes within a species may have the same P content; however, they can vary in P use, which could lead to a different result in terms of growth and excretion rates. For example, P allocated to tissues such as the *Daphnia* exoskeleton (i.e. carapace; [26]) should have slow turnover compared to high turnover molecules such as phospholipids [51], thus imparting distinctive signatures on P retention and excretion rates. For this reason, quantifying elemental use (i.e. acquisition, assimilation, net incorporation, retention), particularly when significant variation is present among genotypes, may be more applicable for examining intraspecific variation, and potentially also variation at broader taxonomic levels. While ES has provided new insights into how the balance of elements affects ecological interactions, little is known about evolutionary dynamics in stoichiometric traits [17]. In addition to answering basic questions about the evolutionary genetics of elemental content [52], addressing elemental kinetics is bound to illuminate the evolutionary sources and mechanisms maintaining substantial intraspecific variation in stoichiometry.

## Supplementary Material

Fig. S1

## Supplementary Material

Supplementary information

## Data accessibility

All data used in this manuscript are present in the manuscript and its electronic supplementary material.
