# Supplementary figures and images for: Genotype-specific relationships among phosphorus use, growth and abundance in Daphnia pulicaria
Source: R Soc Open Sci. 2017 Dec 13;4(12):170770. doi: 10.1098/rsos.170770 (PMC5749992; doi:10.1098/rsos.170770)

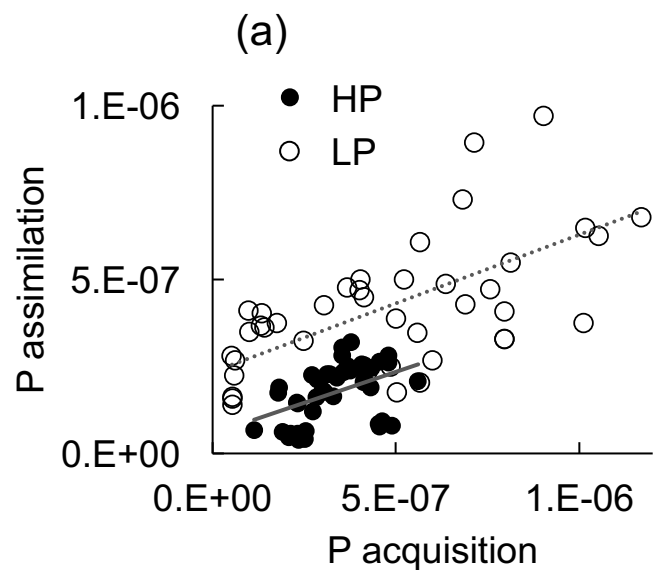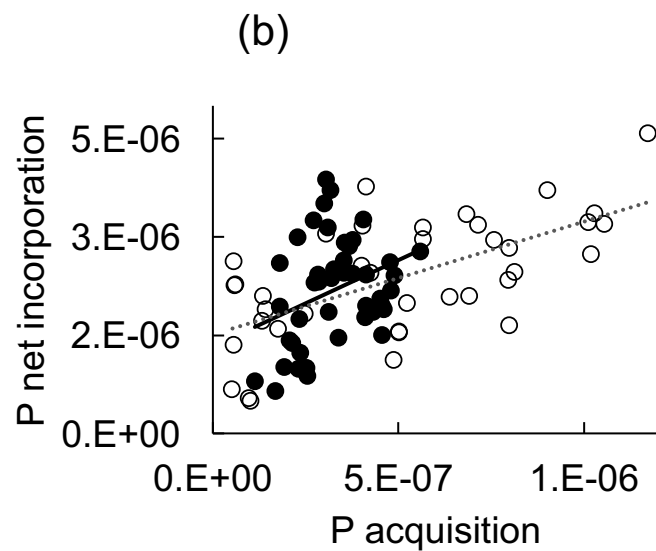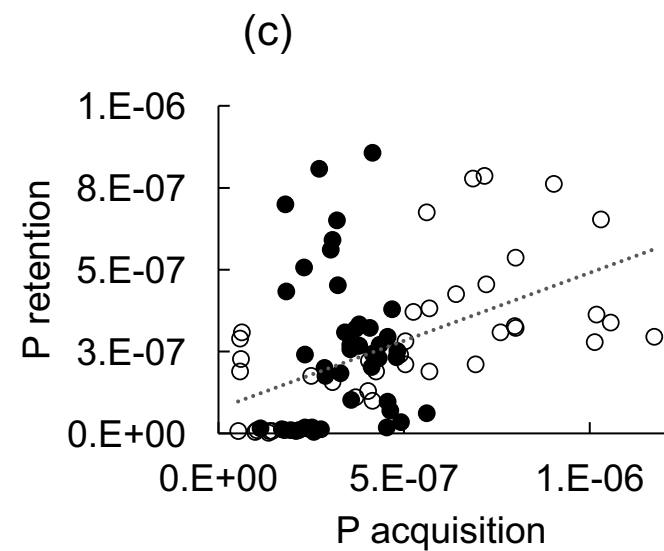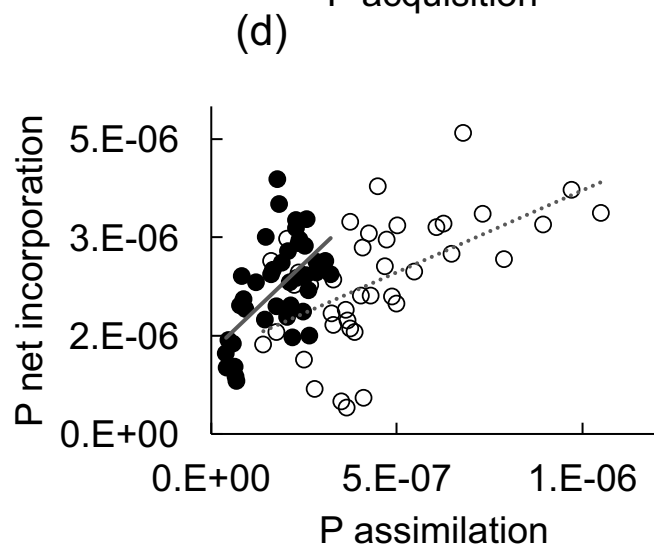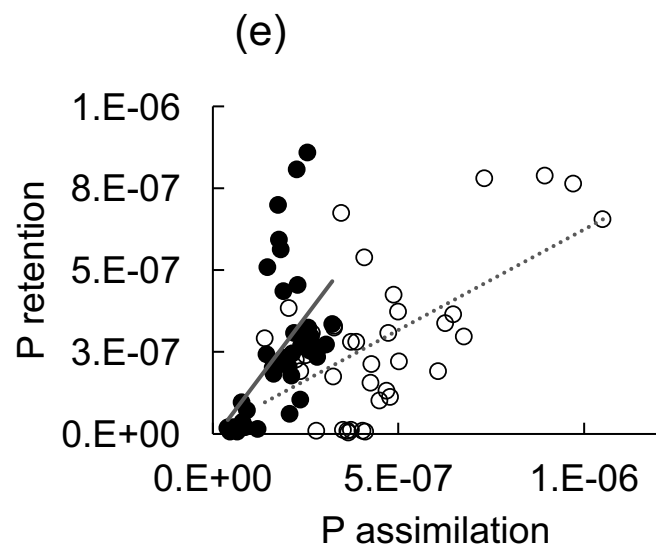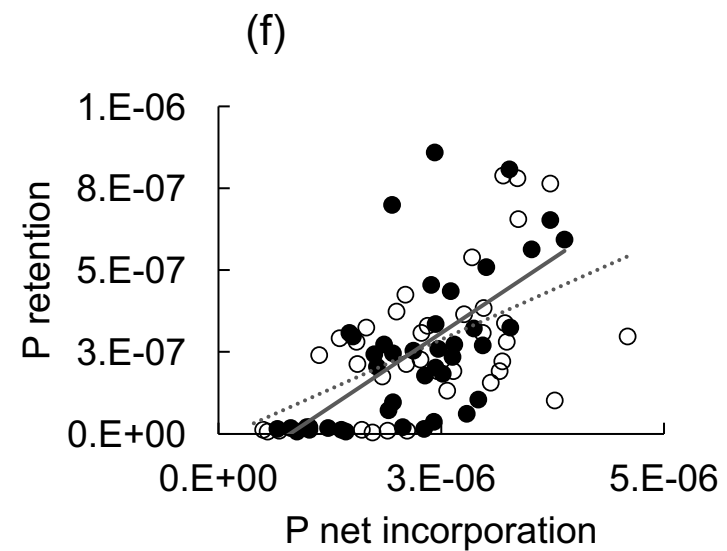

Supplement: Fig. S1 [file rsos170770supp1.pdf]
