# Supplementary material for: Genotype-specific relationships among phosphorus use, growth and abundance in Daphnia pulicaria
Source: R Soc Open Sci. 2017 Dec 13;4(12):170770. doi: 10.1098/rsos.170770 (PMC5749992; doi:10.1098/rsos.170770)
Supplement: Supplementary information [file rsos170770supp2.docx]

**Supplementary information**

**Figure S1** P-use linear relationships. Open circles represent Low Phosphorus (LP treatment; solid circles represent High Phosphorus (HP) treatment. All are significantly correlated (p<0.0001). Solid trend lines show significant relationships in HP, and dotted trend lines show significant relationships in LP. All units are represented in micrograms per milligram dry weight.

**

**

**Table S1.** Descriptive statistics of all the traits measured, including mean, standard deviation, sample size, and number of samples excluded (pairwise), from the multivariate analyses (PCA).

| Descriptive Statistics | | | | |
| --- | --- | --- | --- | --- |
| Trait | Mean | Std. Deviation | Analysis N | Missing N |
| Growth rate | 0.056 | 0.040 | 80 | 16 |
| Competitive ability | 0.062 | 0.018 | 48 | 48 |
| Density | 15.312 | 10.373 | 48 | 48 |
| P content | 0.123 | 0.030 | 53 | 43 |
| P acquisition | <0.001 | <0.001 | 91 | 5 |
| P retention | <0.001 | <0.001 | 85 | 11 |
| P net incorporation | 0.002 | <0.001 | 86 | 10 |
| P assimilation | <0.001 | <0.001 | 87 | 9 |

**Table S2.** Principal component analysis of *Daphnia pulicaria* responses to altered P supply conditions.

| Rotated Component Matrix | | | |
| --- | --- | --- | --- |
| Trait | Component | | |
|  | 1 | 2 | 3 |
| P acquisition | 0.883 | -0.039 | -0.074 |
| P assimilation | 0.806 | -0.331 | 0.294 |
| P net incorporation | 0.766 | 0.367 | 0.107 |
| P retention | 0.781 | 0.366 | 0.007 |
| Competitive ability | 0.006 | 0.905 | 0.209 |
| Growth rate | 0.019 | 0.775 | -0.332 |
| Density | 0.271 | 0.522 | 0.287 |
| P content | 0.053 | 0.066 | 0.916 |
| Total Variance Explained | | | |
| Component | Eigenvalue | % of Variance | Cumulative % |
| 1 | 3.002 | 33.781 | 33.781 |
| 2 | 1.867 | 25.958 | 59.739 |
| 3 | 1.090 | 14.745 | 74.485 |

**Table S3** Model selection table with competitive ability as dependent variable.

| P acq. | P assim. | P net. | P ret. | P content | df | Log Lik | AICc | ΔAIC | weight |
| --- | --- | --- | --- | --- | --- | --- | --- | --- | --- |
|  | 477500 |  | -511000 |  | 4 | 2.592 | 3.9 | 0.00 | 0.231 |
|  | 412100 | -128700 |  |  | 4 | 2.064 | 5.0 | 1.06 | 0.136 |
|  | 498200 | -67710 | -341200 |  | 5 | 3.262 | 5.1 | 1.24 | 0.124 |
|  | 515300 |  | -538100 | -0.22720 | 5 | 2.696 | 6.3 | 2.38 | 0.070 |
| -46000 | 506000 |  | -500100 |  | 5 | 2.618 | 6.4 | 2.53 | 0.065 |
|  |  | -86010 |  |  | 3 | -0.412 | 7.5 | 3.56 | 0.039 |
| 35110 | 391400 | -132100 |  |  | 5 | 2.077 | 7.5 | 3.61 | 0.038 |
|  | 415400 | -129200 |  | -0.02435 | 5 | 2.065 | 7.5 | 3.64 | 0.037 |
|  |  |  | -302500 |  | 3 | -0.494 | 7.6 | 3.72 | 0.036 |
|  | 536700 | -67870 | -368300 | -0.23060 | 6 | 3.373 | 7.7 | 3.76 | 0.035 |
| 33520 | 478400 | -70940 | -341000 |  | 6 | 3.275 | 7.8 | 3.95 | 0.032 |
| 256400 |  | -126200 |  |  | 4 | 0.554 | 8.0 | 4.08 | 0.030 |
| 208700 |  |  | -408500 |  | 4 | 0.194 | 8.7 | 4.80 | 0.021 |
| -92570 | 587000 |  | -526300 | -0.31220 | 6 | 2.788 | 8.8 | 4.93 | 0.020 |
|  |  |  |  |  | 2 | -2.352 | 9.0 | 5.11 | 0.018 |
|  |  | -52360 | -164100 |  | 4 | -0.147 | 9.4 | 5.48 | 0.015 |
|  |  | -83430 |  | 0.34460 | 4 | -0.159 | 9.4 | 5.50 | 0.015 |
| 287600 |  | -85320 | -223200 |  | 5 | 1.055 | 9.6 | 5.66 | 0.014 |
|  |  |  | -289000 | 0.29110 | 4 | -0.317 | 9.7 | 5.82 | 0.013 |
| 269700 |  | -125200 |  | 0.40450 | 5 | 0.918 | 9.8 | 5.93 | 0.012 |

**Table S4** Model selection table with growth rate as the dependent variable.

| P acq. | P assim. | P net. | P ret. | P content | df | Log lik | AICc | ΔAIC | weight |
| --- | --- | --- | --- | --- | --- | --- | --- | --- | --- |
| 52550 | -128900 |  | 67660 |  | 5 | 84.076 | -156.5 | 0.00 | 0.230 |
|  | -96270 |  | 80090 |  | 4 | 82.483 | -155.9 | 0.06 | 0.170 |
|  | -86340 | 20360 |  |  | 4 | 81.988 | -154.9 | 1.59 | 0.104 |
|  | -99630 | 10950 | 52630 |  | 5 | 83.272 | -154.9 | 1.61 | 0.103 |
| 44910 | -112900 | 16070 |  |  | 5 | 82.987 | -154.3 | 2.18 | 0.077 |
| 45150 | -126400 | 6599 | 52860 |  | 6 | 84.349 | -154.3 | 2.19 | 0.077 |
| 57420 | -137400 |  | 70390 | 0.03262 | 6 | 84.166 | -153.9 | 2.55 | 0.064 |
|  | -92920 |  | 77700 | -0.02008 | 5 | 82.519 | -153.4 | 3.11 | 0.048 |
|  | -79800 | 19320 |  | -0.04772 | 5 | 82.204 | -152.7 | 3.74 | 0.035 |
|  | -96370 | 10940 | 50340 | -0.01953 | 6 | 83.308 | -152.2 | 4.27 | 0.027 |
| 74760 | -108000 |  |  |  | 4 | 80.579 | -152.1 | 4.41 | 0.025 |
| 43060 | -110300 | 16010 |  | -0.01111 | 6 | 82.997 | -151.6 | 4.89 | 0.020 |
| 49410 | -133100 | 6206 | 55880 | 0.02558 | 7 | 84.403 | -151.5 | 4.97 | 0.019 |
